# Supplementary material for: Vernicia fordii leaf extract inhibited anthracnose growth by downregulating reactive oxygen species (ROS) levels in vitro and in vivo
Source: PeerJ. 2024 Jul 22;12:e17607. doi: 10.7717/peerj.17607 (PMC11271649; doi:10.7717/peerj.17607)
Supplement: Supplemental Information 1 — GAPDH is the reference gene. [file peerj-12-17607-s001.docx]

| Gene name | Forward Primer（5′-3′） | Reverse Primer（5′-3′） | Reference |
| --- | --- | --- | --- |
| *Mkk1* | GAATCCCGATCCCGACGTTAAGAAG | CGAGATGGTGCCAGTAGTGTTGTC | Xiao et al., 2021 |
| *HAC1* | CAGATGCCGAGGACAACGAAGTG | CCAGCCATGATTGCGGAAGGTC | Yao, Q. et al., 2019 |
| *Rab7* | ACAACTTCCCATTCGTCGTCCTTG | TAGCCTCCTTCGCACTGGTCTC | Wu Yongyi et al., 2022 |
| *VAM7* | GCAGATGATGAGCGAGCAGGAC | CGCCTCCACCTCGTCATTGATC | Li, S.Z. et al., 2021 |
| *VPS39* | CGAAGAGACCGACAGCATCAAGG | GTAGGCGTGTTCTCGTTCCAACC | Li, S.Z. et al., 2021 |
| *GAPDH* | GCCGTCAACGACCCCTTCATTGA | GGGTGGAGTCGTACTTGAGCATGT | Lingxue Cao et al., 2023 |
