# Supplementary material for: Vernicia fordii leaf extract inhibited anthracnose growth by downregulating reactive oxygen species (ROS) levels in vitro and in vivo
Source: PeerJ. 2024 Jul 22;12:e17607. doi: 10.7717/peerj.17607 (PMC11271649; doi:10.7717/peerj.17607)
Supplement: Supplemental Information 6 [file peerj-12-17607-s006.docx]

1. Experimental Design

The qPCR assay in this study consisted of a control group and an experimental group. The control group was cultivated with the *Colletotrichum fructicola* in a PDB liquid medium. The experimental group was involved in the addition of different concentrations of *Vernicia fordii* leaf extract (VFLE) to a PDB liquid medium, followed by cultivation with *C. fructicola*. Each group included three replicates, and the experiment was repeated three times.

2. Sample (Line 164-Line 171)

The PDB medium was supplemented with VFLE at final concentrations of 0, 100, 200, 400, and 600 µg/mL, followed by the addition of 6 mm mycelial discs. Cultures were incubated at 28°C and 160 rpm for 48h until mycelial growth was observed. Following this, mycelial blocks from the conical flasks were aseptically collected using sterilized forceps after high-temperature sterilization and cooling. The collected mycelia were rapidly ground using liquid nitrogen in a grinding bowl. The resulting powdered samples were individually transferred into RNase/RDnase-free 1.5 mL centrifuge tubes and temporarily stored at -80°C.

3. Nucleic acid extraction (Line 164-Line 171)

Following the powderization, RNA extraction of the samples was immediately conducted using the RNAprep Pure Plant Total RNA Extraction Kit. The extracted RNA samples were labeled as CK A, 0.5A, 1A, 2A, and 3A, respectively. The extraction procedure was carried out in strict accordance with the instructions provided. Subsequently, RNA samples were subjected to electrophoresis for analysis, and the electrophoresis map showed that the RNA was successfully extracted. The RNA samples were stored at -80°C.


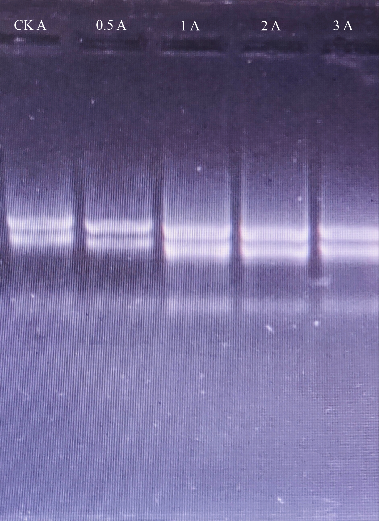


Fig 1. RNA electrophoresis map

4. Reverse transcription

The Evo M-MLV RT Kit with gDNA Clean for qPCR (AG) was utilized for reverse transcription. The resulting cDNA was stored at -20°C. The experimental procedure consisted of two steps, as illustrated in the following table:

Table 1. DNA remove reaction system

| Element | Volume |
| --- | --- |
| gDNA Clean Reagent | 1μL |
| 5× gDNA Clean Buffer | 2μL |
| Total RNA | 1μL |
| RNase free water | 6μL |

Reaction condition：42℃ for 2min

4℃

Table 2. Preparation of cDNA synthesis reaction liquid line

| Element | Volume |
| --- | --- |
| Step (1) reaction liquid | 10μL |
| RNase free water | 3μL |
| 5× RTase Reaction Buffer Mix I | 4μL |
| Oligo Dt(18T) Primer (50μM) | 1μL |
| Rnadom 6 mers Primer (400μM) | 1μL |
| *Evo M-MLV* RTase Enzyme Mix | 1μL |
| Total | 20μL |

Reaction condition：37℃ for 15min

85℃ for 5sec

4℃

5. qPCR oligonucleotides (Line 168-Line 169)

Genes that affect the pathogenicity of *C. fructicola* were identified from a relevant research article. The gene sequences as described in the paper were submitted to Sangon Biotech for primer design to be used in subsequent experiments.

Table 3. qPCR primers described

| Gene name | Forward Primer（5′-3′） | Reverse Primer（5′-3′） |
| --- | --- | --- |
| Mkk1 | GAATCCCGATCCCGACGTTAAGAAG | CGAGATGGTGCCAGTAGTGTTGTC |
| HAC1 | CAGATGCCGAGGACAACGAAGTG | CCAGCCATGATTGCGGAAGGTC |
| Rab7 | ACAACTTCCCATTCGTCGTCCTTG | TAGCCTCCTTCGCACTGGTCTC |
| VAM7 | GCAGATGATGAGCGAGCAGGAC | CGCCTCCACCTCGTCATTGATC |
| VPS39 | CGAAGAGACCGACAGCATCAAGG | GTAGGCGTGTTCTCGTTCCAACC |
| GAPDH | GCCGTCAACGACCCCTTCATTGA | GGGTGGAGTCGTACTTGAGCATGT |

Note: *GAPDH* is a reference gene.

6. qPCR protocol (Line 169-Line 170)

The SYBR Green Premix Pro Taq HS qPCR Kit purchased (AG) was employed for the qPCR experiment. The qPCR instrument used was the CFX96 Touch Real-Time PCR Detection System. Each sample was run in triplicate, and the experiment was conducted with three replicates. The specific operational procedures were as follows:

Table 4. qPCR reaction system

| Element | Volume |
| --- | --- |
| 2X SYBR Green Pro Taq HS Premix | 10μL |
| Template | 1μL |
| Primer F (10μM) | 0.4μL |
| Primer R (10μM) | 0.4μL |
| RNase free water | 8.2μL |
| Total | 20μL |

Table 5. qPCR reaction condition

|  | Temperature | Times | Cycles |
| --- | --- | --- | --- |
| Step 1 | 95℃ | 30sec | 1 |
| Step 2 | 95℃  60℃ | 5sec  30sec | 40 |
| Step 3 | 72℃ | 10min | 1 |

7. Date analysis (Line 187-Line 189)

Table 6. The Cq values of each sample

| Sample | Target | Cq |
| --- | --- | --- |
| CK A | *GAPDH* | 26.84 |
| CK A | GAPDH | 26.81 |
| CK A | GAPDH | 26.76 |
| 0.5A | GAPDH | 29.56 |
| 0.5A | GAPDH | 30.10 |
| 0.5A | GAPDH | 28.79 |
| 1A | GAPDH | 21.45 |
| 1A | GAPDH | 21.23 |
| 1A | GAPDH | 21.33 |
| 2A | GAPDH | 22.43 |
| 2A | GAPDH | 22.26 |
| 2A | GAPDH | 22.25 |
| 3A | GAPDH | 30.65 |
| 3A | GAPDH | 30.43 |
| 3A | GAPDH | 30.29 |
| CK A | HAC1 | 26.22 |
| CK A | HAC1 | 26.09 |
| CK A | HAC1 | 26.13 |
| 0.5A | HAC1 | 28.71 |
| 0.5A | HAC1 | 28.32 |
| 0.5A | HAC1 | 28.55 |
| 1A | HAC1 | 25.58 |
| 1A | HAC1 | 25.78 |
| 1A | HAC1 | 25.97 |
| 2A | HAC1 | 25.78 |
| 2A | HAC1 | 25.88 |
| 2A | HAC1 | 25.82 |
| 3A | HAC1 | 31.85 |
| 3A | HAC1 | 31.73 |
| 3A | HAC1 | 31.59 |
| CK A | Mkk1 | 27.32 |
| CK A | Mkk1 | 27.16 |
| CK A | Mkk1 | 27.24 |
| 0.5A | Mkk1 | 28.08 |
| 0.5A | Mkk1 | 28.14 |
| 0.5A | Mkk1 | 28.33 |
| 1A | Mkk1 | 23.98 |
| 1A | Mkk1 | 24.10 |
| 1A | Mkk1 | 24.16 |
| 2A | Mkk1 | 24.24 |
| 2A | Mkk1 | 24.83 |
| 2A | Mkk1 | 24.32 |
| 3A | Mkk1 | 29.42 |
| 3A | Mkk1 | 29.44 |
| 3A | Mkk1 | 29.75 |
| CK A | Rab7 | 22.07 |
| CK A | Rab7 | 22.43 |
| CK A | Rab7 | 22.01 |
| 0.5A | Rab7 | 22.32 |
| 0.5A | Rab7 | 22.49 |
| 0.5A | Rab7 | 22.40 |
| 1A | Rab7 | 20.56 |
| 1A | Rab7 | 20.55 |
| 1A | Rab7 | 20.59 |
| 2A | Rab7 | 23.26 |
| 2A | Rab7 | 23.45 |
| 2A | Rab7 | 23.32 |
| 3A | Rab7 | 28.13 |
| 3A | Rab7 | 28.23 |
| 3A | Rab7 | 28.43 |
| CK A | VAM7 | 24.06 |
| CK A | VAM7 | 24.54 |
| CK A | VAM7 | 24.12 |
| 0.5A | VAM7 | 26.18 |
| 0.5A | VAM7 | 25.86 |
| 0.5A | VAM7 | 25.74 |
| 1A | VAM7 | 23.96 |
| 1A | VAM7 | 24.06 |
| 1A | VAM7 | 23.86 |
| 2A | VAM7 | 25.19 |
| 2A | VAM7 | 25.79 |
| 2A | VAM7 | 25.23 |
| 3A | VAM7 | 26.97 |
| 3A | VAM7 | 26.93 |
| 3A | VAM7 | 26.87 |
| CK A | VPS39 | 24.52 |
| CK A | VPS39 | 24.59 |
| CK A | VPS39 | 24.23 |
| 0.5A | VPS39 | 25.30 |
| 0.5A | VPS39 | 25.68 |
| 0.5A | VPS39 | 24.89 |
| 1A | VPS39 | 24.19 |
| 1A | VPS39 | 24.47 |
| 1A | VPS39 | 24.34 |
| 2A | VPS39 | 25.67 |
| 2A | VPS39 | 25.85 |
| 2A | VPS39 | 25.50 |
| 3A | VPS39 | 28.29 |
| 3A | VPS39 | 28.45 |
| 3A | VPS39 | 28.65 |

Note: *GAPDH* is a reference gene.

The 2-ΔΔCt method was employed to analyze the Cq values, and SPSS software was used to analyze the intra-group differences among the samples.
