# Supplementary figures and images for: Vernicia fordii leaf extract inhibited anthracnose growth by downregulating reactive oxygen species (ROS) levels in vitro and in vivo
Source: PeerJ. 2024 Jul 22;12:e17607. doi: 10.7717/peerj.17607 (PMC11271649; doi:10.7717/peerj.17607)

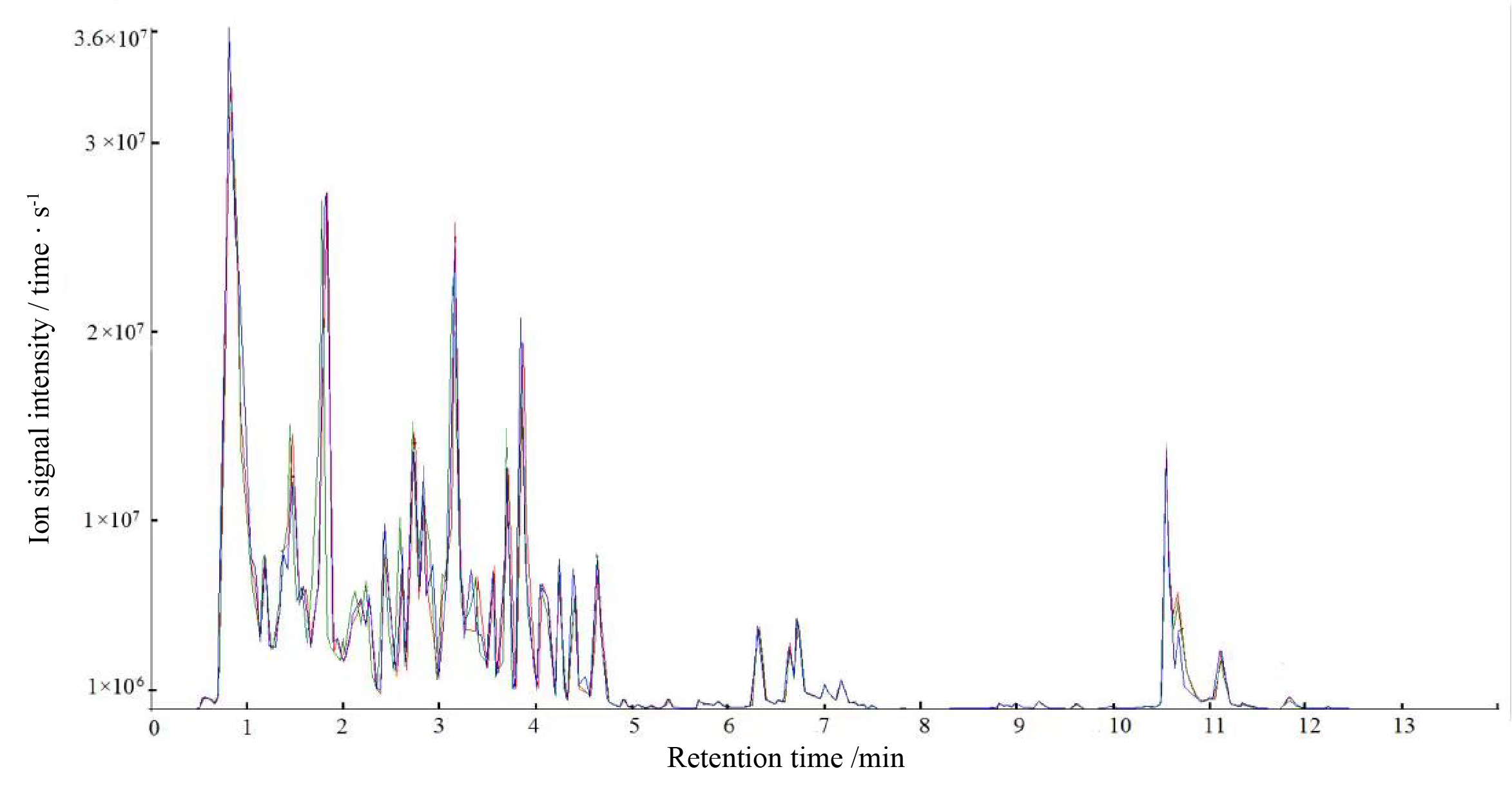

Supplement: Supplemental Information 7 [file peerj-12-17607-s007.png]
